# Supplementary figures and images for: Chicago sky blue gel for better visualization of Demodex in patients with Demodex blepharitis
Source: PeerJ. 2023 Nov 17;11:e16378. doi: 10.7717/peerj.16378 (PMC10658889; doi:10.7717/peerj.16378)

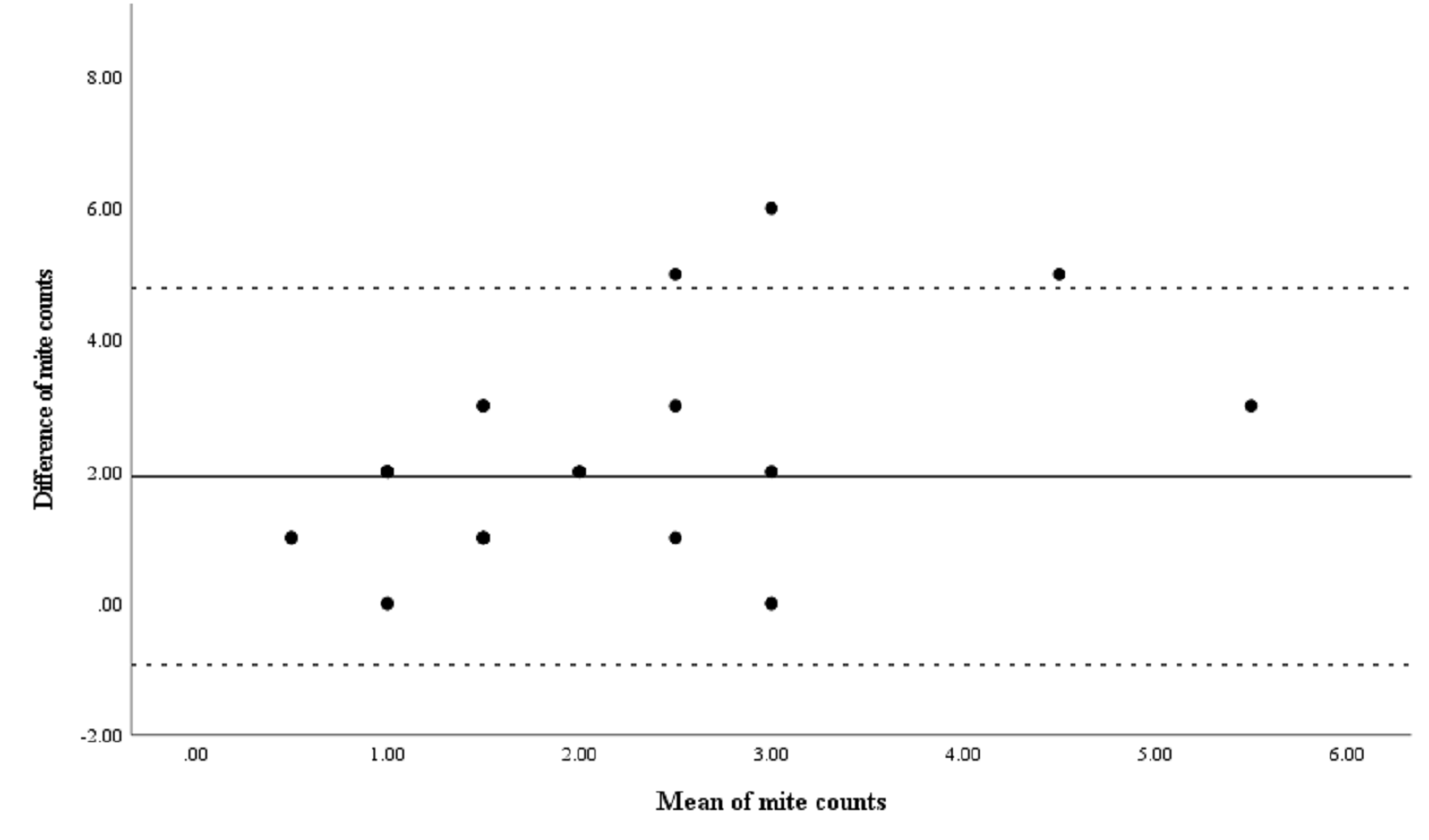

Supplement: Figure S1 — Each dot represents one data point. The concordance between these diagnostic tests was examined using 1.96SD (95% CI) represented by solid line. [file peerj-11-16378-s002.png]

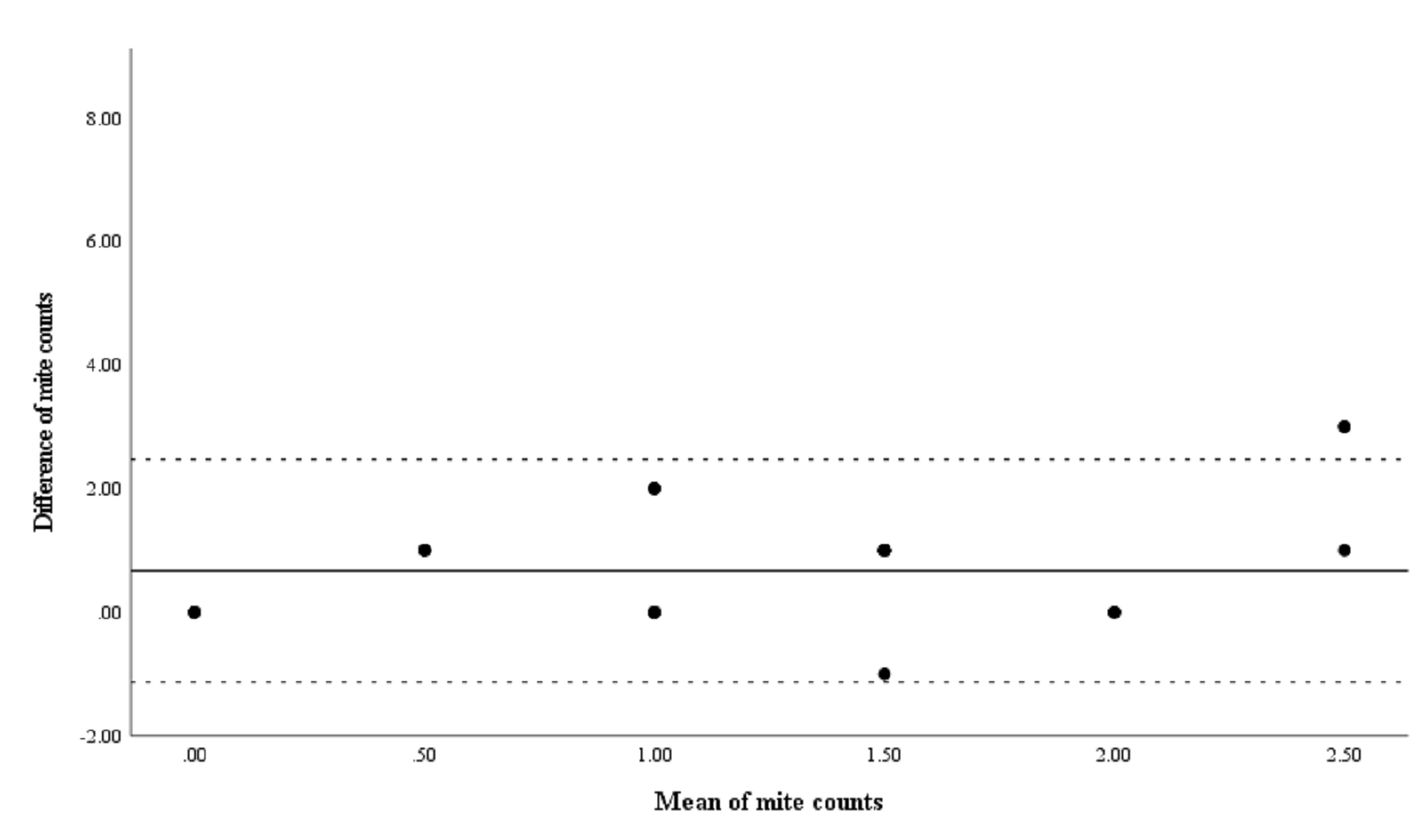

Supplement: Figure S2 — Each dot represents one data point. The concordance between these diagnostic tests was examined using 1.96SD (95% CI) represented by solid line. [file peerj-11-16378-s003.png]

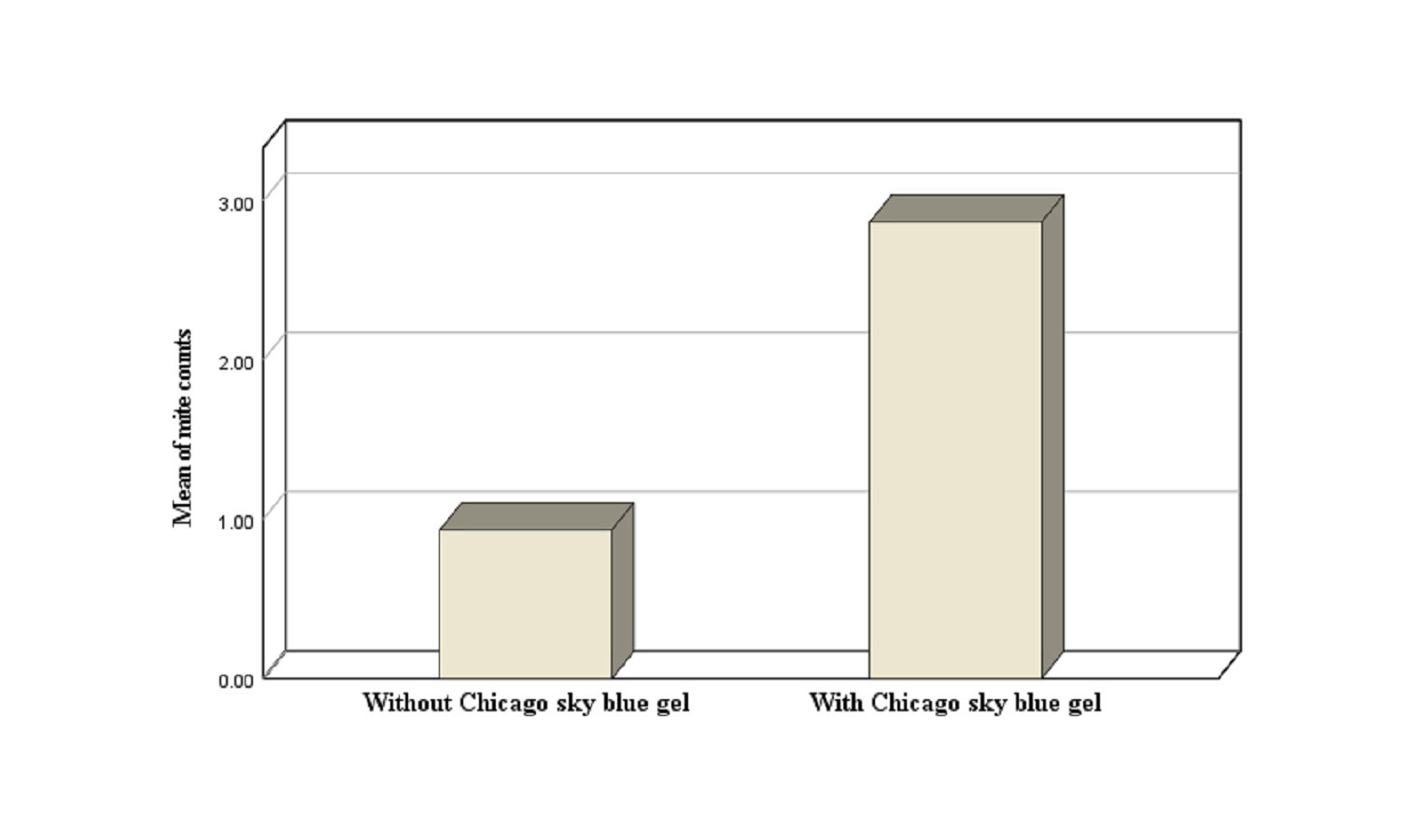

Supplement: Figure S3 — The mean number of mites using light microscopy with and without Chicago sky blue gel was 0.93 (SD 1.08) and 2.87 (SD 1.63), respectively. [file peerj-11-16378-s004.png]

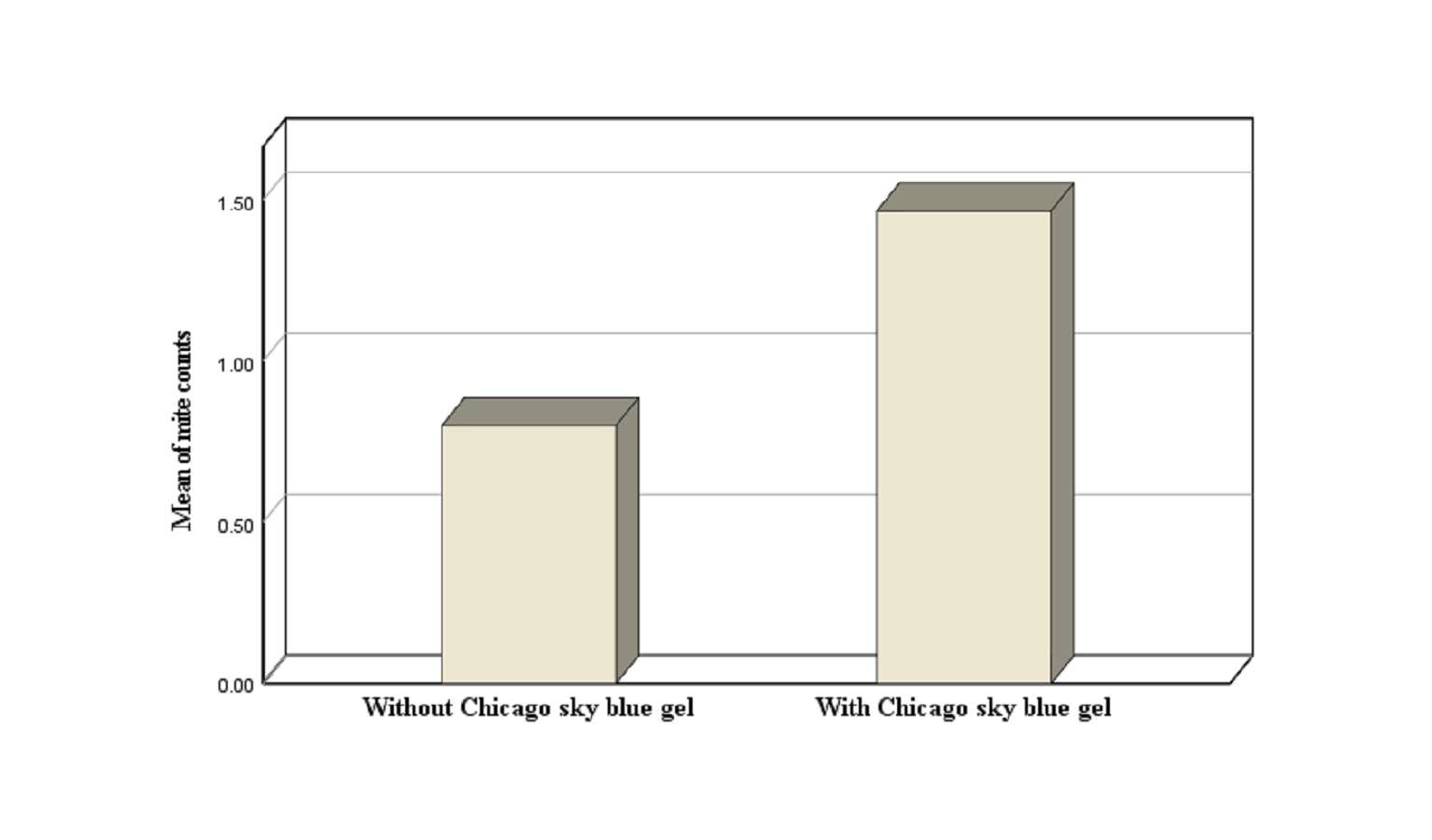

Supplement: Figure S4 — The mean number of mites using light microscopy with and without Chicago sky blue gel was 0.80 (SD 0.66) and 1.47 (SD 1.04), respectively. [file peerj-11-16378-s005.png]
